# Transplantation of the Cornea

**Published:** 1879-09

**Authors:** 


### TRANSPLANTATION OF THE CORNEA.

The literature of this subject is extensive, considering the short time since systematic attempts were first made to accomplish the much desired effect. The experiments of Von Hippel gave an impetus to the study of the operation, which has resulted in a series of excellent articles concerning it by Duerr, Rosmini, Schoeler and others. Their object has been to improve the condition of that large class of unfortunates whose cornea have become opaque, by substituting that of a dog, or other animal. The idea is plausible, and when the apparent difficulties are taken into account, it is remarkable how readily the transplanted portion will adhere in its new situation. The main obstacle to success, however, is the tendency of the engrafted tissue to become subsequently opaque. Several ingenious procedures have been resorted to, with a view to obviate this, by increasing the vascular supply, and thus rendering the nutrition of the part more nearly normal. For example, one operator has adopted a plan about as follows: Around the cornea of the animal, from which the piece is to be taken, a number of small wounds or ulcers are artificially produced. The adjacent conjunctiva being then drawn forward and stretched over these spots, it is retained there by means of sutures, and in a short time becomes permanently adherent. In this way, a circle of growths is formed about the centre of the cornea, each one of them being a veritable pterygium, like that so often seen in the inner angle of the eye. The central, clear portion of cornea, with the attached conjunctiva, is then cut out, and a corresponding piece having been already removed from the diseased human eye, the fragment is secured in its new position by means of several sutures. By this, and by similar procedures, the transplanted portion has frequently been made to attach itself firmly—although, as previously remarked, the piece soon tends to become opaque.

In Prof. Hippel's last contribution on the subject, he reports five cases. One of these proved a complete failure, three could recognize large objects like the hand immediately before the eye, and one, for a time, could count fingers at eight inches.

This is not a very brilliant showing, it is true; but when we remember that until now such cases have been considered hopelessly blind, and when we realize further how much each additional ray of light is valued by such unfortunates, it must already be counted as a warrantable operation, as a procedure by which nothing can be lost, but considerable may be gained, and promises in the future to become one of the triumphs of ophthalmic surgery.

